# Higher Atmospheric CO_2_ Levels Favor C_3_ Plants Over C_4_ Plants in Utilizing Ammonium as a Nitrogen Source

**DOI:** 10.3389/fpls.2020.537443

**Published:** 2020-12-02

**Authors:** Feng Wang, Jingwen Gao, Jean W. H. Yong, Qiang Wang, Junwei Ma, Xinhua He

**Affiliations:** ^1^Institute of Environmental Resources, Soil and Fertilizer, Zhejiang Academy of Agricultural Sciences, Hangzhou, China; ^2^Centre of Excellence for Soil Biology, College of Resources and Environment, Southwest University, Chongqing, China; ^3^School of Biological Sciences, The University of Western Australia, Perth, WA, Australia; ^4^Department of Biosystems and Technology, Swedish University of Agricultural Sciences, Alnarp, Sweden

**Keywords:** atmospheric CO_2_, ecophysiology, electron transport, NH_4_^+^ stress, photosynthesis, *Triticum aestivum*, Zea mays

## Abstract

Photosynthesis of wheat and maize declined when grown with NH_4_^+^ as a nitrogen (N) source at ambient CO_2_ concentration compared to those grown with a mixture of NO_3_^–^ and NH_4_^+^, or NO_3_^–^ as the sole N source. Interestingly, these N nutritional physiological responses changed when the atmospheric CO_2_ concentration increases. We studied the photosynthetic responses of wheat and maize growing with various N forms at three levels of growth CO_2_ levels. Hydroponic experiments were carried out using a C_3_ plant (wheat, *Triticum aestivum* L. cv. Chuanmai 58) and a C_4_ plant (maize, *Zea mays* L. cv. Zhongdan 808) given three types of N nutrition: sole NO_3_^–^ (NN), sole NH_4_^+^ (AN) and a mixture of both NO_3_^–^ and NH_4_^+^ (Mix-N). The test plants were grown using custom-built chambers where a continuous and desired atmospheric CO_2_ (*C*_*a*_) concentration could be maintained: 280 μmol mol^–1^ (representing the pre-Industrial Revolution CO_2_ concentration of the 18th century), 400 μmol mol^–1^ (present level) and 550 μmol mol^–1^ (representing the anticipated futuristic concentration in 2050). Under AN, the decrease in net photosynthetic rate (*P*_*n*_) was attributed to a reduction in the maximum RuBP-regeneration rate, which then caused reductions in the maximum Rubisco-carboxylation rates for both species. Decreases in electron transport rate, reduction of electron flux to the photosynthetic carbon [*Je(PCR)*] and electron flux for photorespiratory carbon oxidation [*Je(PCO)*] were also observed under AN for both species. However, the intercellular (*C*_*i*_) and chloroplast (*C*_*c*_) CO_2_ concentration increased with increasing atmospheric CO_2_ in C_3_ wheat but not in C_4_ maize, leading to a higher *Je(PCR)/ Je(PCO)* ratio. Interestingly, the reduction of *P*_*n*_ under AN was relieved in wheat through higher CO_2_ levels, but that was not the case in maize. In conclusion, elevating atmospheric CO_2_ concentration increased *C*_*i*_ and *C*_*c*_ in wheat, but not in maize, with enhanced electron fluxes towards photosynthesis, rather than photorespiration, thereby relieving the inhibition of photosynthesis under AN. Our results contributed to a better understanding of NH_4_^+^ involvement in N nutrition of crops growing under different levels of CO_2_.

## Introduction

The application of chemical nitrogen (N) fertilizers has greatly increased global crop yields and decreased world hunger over the past five decades (Gong et al., 2011). However, only 30–40 % of applied N is utilized by crops; most is lost in numerous ways, including run-off, leaching, denitrification and volatilization, which together lead to a range of environmental problems (Richter and Roelcke, 2000; Xing and Zhu, 2000). Thus, increasing plant nitrogen use efficiency (NUE) is crucial for the development of sustainable agriculture. Unlike nitrate (NO_3_^–^), ammonium (NH_4_^+^) can be assimilated by plants without further chemical reduction (Mehrer and Mohr, 1989; Onoda et al., 2004). NH_4_^+^ can be provided by both manure and urea fertilizers (Xu et al., 2012; Coskun et al., 2017). A promising future strategy for improving agronomic NUE is the application of stabilized-NH_4_^+^-based fertilizers together with other active compounds, such as nitrification inhibitors, which can inhibit the nitrification of NH_4_^+^, thereby maintaining a high soil N content in the form of NH_4_^+^ over extended periods (IPCC, 2007; Ariz et al., 2011). Some crops, including wheat and maize, are able to grow well when provided with a mixture of NO_3_^–^ and NH_4_^+^ (Mix-N), or NO_3_^–^ as the sole N source (NN) (Miller and Cramer, 2005). Under certain environmental conditions, NH_4_^+^ may reduce growth by decreasing photosynthesis, thereby lowering crop productivity (Britto and Kronzucker, 2002). Since urea and NH_4_^+^-based N fertilizers are used commonly to support the growth of cereals, vegetables and fruits, a better understanding of the toxic effects of NH_4_^+^ in plant nutrition should facilitate better crop productivity (Miller and Cramer, 2005; Fernández-Crespo et al., 2012).

Photosynthesis is a synergistic process that involves electron harvesting, transport, and utilization (Kühlbrandt et al., 1994). Photosynthetic electron transport typically involves two reaction centers: photosystems I and II (PSI and PSII, respectively). The D1 protein of PSII is sensitive to NH_4_^+^, and a loss of PSII function occurs when NH_4_^+^-based fertilizers are applied in excessive quantities (Drath et al., 2008). When this occurs, impairment of the photosynthetic electron transport chain will lead to a decrease in photochemical efficiency (*Φ_*PSII*_*) and the electron transport rate (*J*_*t*_), in turn leading to a deficiency in NADPH and ATP for CO_2_ assimilation (Wang et al., 2019). The atmospheric CO_2_ concentration (*C*_*a*_) has increased from 280 μmol mol^–1^ in pre-industrial times to 400 μmol mol^–1^ at present, and is expected to reach 550 μmol mol^–1^ by the 2050 s (IPCC, 2013). The elevation of CO_2_ concentration at the sites of Rubisco carboxylation alters plant photosynthetic sensitivity, potentially modulating sensitivity to a diversity of N sources. Due to the importance of food production security and crops in the global carbon cycle, an improved understanding of *C*_*a*_ changes on the N nutrition of C_3_ and C_4_ plants will become more and more crucial (Lloyd and Farquhar, 1996; Ghannoum et al., 2000). In general, plants with the C_4_ photosynthetic pathway have anatomical and biochemical traits that increase CO_2_ levels around the carboxylating Rubisco enzyme (Hatch and Slack, 1966). Bloom et al. (2014) and Dier et al. (2018) showed that NO_3_^–^ assimilation is inhibited by elevated CO_2_ concentrations in field-grown C_3_ wheat plants. We postulated that an elevated *C*_*a*_ increases photosynthesis to produce more carbon skeletons, which in turn would increase NH_4_^+^ assimilation, thereby ameliorating the possible physiological stress of having excess free NH_4_^+^ in C_3_ plants. The detailed comparative physiological responses of C_3_ and C_4_ plants to NH_4_^+^ fertilization under elevated *C*_*a*_ are still not fully understood (Bloom et al., 2002, 2010; Cousins and Bloom, 2003; Bloom, 2015).

A wide variety of equipment, including open-top chambers (OTC), controlled-environment (CE) systems, and free-air CO_2_ enrichment (FACE) systems, has been used to study the effects of elevated *C*_*a*_. In the OTC system, plants are held in a chamber with an open top that facilitates gas exchange with the atmosphere (Owensby et al., 1999). However, the temperature is generally higher inside the chamber than outside, inevitably increasing plant transpiration, which influences plant growth rates. The FACE system minimally perturbs the plant growth environment and is suitable for long-term experiments under elevated *C*_*a*_. However, the FACE system could not be used to study the effects of sub-ambient CO_2_ concentrations. Controlled environment experiments can also be performed in greenhouses and artificial growth chambers (Yong et al., 2000: Aranjuelo et al., 2009; Kanemoto et al., 2009; Robredo et al., 2011). To design a system having minimal impact on natural temperature, light and seasonality, while also having the capacity to provide either sub-ambient or elevated levels of CO_2_, we custom-fabricated experimental chambers in which the inside temperature and humidity were kept similar to outside open field conditions while regulating the *C*_*a*_.

Our primary objective was to improve understanding of (i) the physiological mechanisms underlying the photosynthetic inhibition caused by NH_4_^+^ nutrition and (ii) the mechanism by which *C*_*a*_ affects the NH_4_^+^ tolerance of C_3_ and C_4_ plants. We therefore studied the photosynthetic responses of a C_3_ plant (wheat, *Triticum aestivum* L.) and a C_4_ plant (maize, *Zea mays* L.). Both these species prefer NO_3_^–^ as the N nutrient source and we grew them under three *C*_*a*_ concentrations (280, 400, or 550 μmol mol^–1^) combined with three forms of N nutrition: Mix-N, NN and sole NH_4_^+^ nitrogen (AN). Our other objective was to provide new knowledge facilitating (i) directed breeding programs aiming to produce N-efficient cultivars, and (ii) the development of sustainable crop N management strategy to adapt to a future with elevated *C*_*a*_, as predicted by current models of global climate change.

## Materials and Methods

### Plant Materials and Experimental Design

**FIGURE 1:**
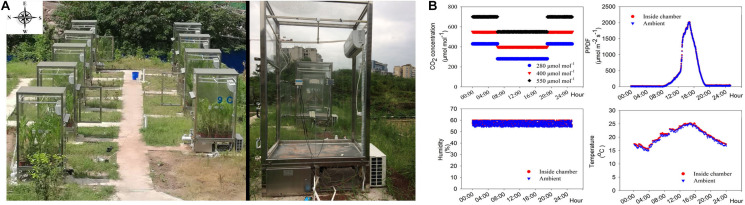
Custom-built chambers **(A)** that automatically maintained desired CO_2_ levels, while temperature and humidity of the chambers were similar to the outside atmospheric environment and **(B)** mean CO_2_ concentrations maintained inside the CO_2_ chamber; intensity of photon flux intensity (PPFD); humidity and temperature inside and outside the chambers over a period of 21 days of growth.

Wheat (*T*. *aestivum* cv. Chuanmai 58) and maize (*Z*. *mays* cv. Zhongdan 808), two common crop species in Chongqing, China, were grown under hydroponic experimental conditions. Seeds of both species, of uniform size, were sterilized in 20% (v/v) H_2_O_2_ for 10 min, rinsed with distilled water, and germinated in darkness in culture dishes covered with wet sterile gauze. When the cotyledons were 1.0 cm long, the seedlings were transferred to silica sand (previously soaked in 1% HCl for 2 days, followed by flushing with copious amounts of water to remove all traces of HCl) and watered twice daily with distilled water. Uniform 14-day-old (two-leaf stage) seedlings were transplanted into opaque plastic growth containers containing a modified Hoagland’s solution (Wang et al., 2016a; Gao et al., 2018), with three N sources: Mix-N, NN or AN. The composition of the Mix-N solution was as follows: macronutrients were provided as 5.0 mM N in the form of Ca(NO_3_)_2_, KNO_3_ and (NH_4_)_2_SO_4_ (the ratio of NO_3_^–^ to NH_4_^+^ in the Mix-N was 3 : 2), 3.0 mM K in the form of KH_2_PO_4_ and KNO_3_, 1.5 mM Ca as Ca(NO_3_)_2_ and CaCl_2_, 1.0 mM Mg as MgSO_4_, 1.0 mM P as KH_2_PO_4_, and 0.6 mM Na as NaCl. Micronutrients were provided as 0.1 mM Fe as Fe-EDTA, 455 × 10^–3^ mM Mn as MnSO_4_, 38.1 × 10^–6^ mM Zn as ZnSO_4_, 15.6 × 10^–6^ mM Cu as CuSO_4_, 2.31 × 10^–3^ mM B as H_3_BO_3_, and 6.2 × 10^–6^ mM Mo as MoO_3_. Macronutrients were provided in the NO_3_^–^-source solution as 5.0 mM N in the form of Ca(NO_3_)_2_ and KNO_3_, 3.0 mM K in the form of KH_2_PO_4_ and KNO_3_, 1.5 mM Ca as Ca(NO_3_)_2_, 1.0 mM Mg as MgSO_4_, 1.0 mM P as KH_2_PO_4_, and 0.5 mM Na as NaCl. Macronutrients were provided in the NH_4_^+^-source solution as 5.0 mM N in the form of (NH_4_)_2_SO_4_, 3.0 mM K as KH_2_PO_4_ and K_2_SO_4_, 1.5 mM Ca as CaCl_2_ and CaSO_4_, 1.0 mM Mg as MgSO_4_, 1.0 mM P as KH_2_PO_4_, and 0.5 mM Na as NaCl. Micronutrients in NN or AN source solution were identical to those in the Mix-N source solution; and then kept in chambers (Figure 1A) with the following CO_2_ levels: 280 μmol mol^–1^ [pre-industrial revolution (i.e., 1840) concentration], 400 μmol mol^–1^ (current level), and 550 μmol mol^–1^ (projected concentration by the 2050 s) (IPCC, 2013). The temperature and humidity inside the chambers were automatically maintained to match those of the atmosphere outside (Figure 1B). During the night, the CO_2_ gradients were held at concentrations 150 ± 1 μmol mol^–1^ above daytime levels (Anderson et al., 2001).

A nitrification inhibitor (dicyandiamide, 1 mM) was added to the nutrition solutions to prevent microbial oxidation of NH_4_^+^. The pH of the Hoagland’s solution was adjusted daily to 5.5 using 0.1 mM NaOH for the plants treated with NH_4_^+^, or with 0.1 mM H_2_SO_4_ for those treated with NO_3_^–^. The N concentrations were kept constant by replacing the media at 3-day intervals; aeration was provided continuously. We used three CO_2_ treatments, with three replicates each in separate chambers (9 chambers in total), and deployed three N treatments for each species in each chamber. Ideally, each experiment should be repeated at least once in every chamber, for each of the planned treatments, to eliminate any intrinsic technical effect of each chamber on plant growth; this represented a potential small shortcoming of the current study in our facility.

### Experimental Field Chambers

The automatically CE facility consisted of a CO_2_ control system (2543CN; Shengsen Corp., Qingdao, China; Supplementary Figures S1-1) and a CO_2_ generator (12864; Shengsen Corp.; Supplementary Figure S1-2). The CO_2_ generator comprised of several components, i.e., an electric connection point pressure meter (STC90C516RD; Shengsen Corp.), pressure sensors (13864; Shengsen Corp.), and Na_2_CO_3_ and H_2_SO_4_ feeding inlets (Supplementary Figures S1-5, 6), where the CO_2_ was generated according to the following equation: Na_2_CO_3_ + H_2_SO_4_ = *Na*_2_SO_4_ + H_2_O + *CO*_2_ The CO_2_ generator was connected to the CO_2_ control system to maintain the CO_2_ concentration in the chambers within a desired set range; CO_2_ was delivered through pipes into each chamber.

The chambers were built using toughened glass (10 mm thick, 99% transmittance) walls and roofs (Supplementary Figure S1-13). The floors were covered using a polyvinyl chloride (PVC) material (Supplementary Figure S1-7). Each chamber measured 1,500 × 1,000 × 2,000 mm (length × width × height). CO_2_, temperature and humidity sensors were mounted on both the outer and inner surfaces of the walls (Supplementary Figures S1-8–10). The environmental control mechanism in this system operated automatically and regulated the internal CO_2_ concentration (±2.5 μmol mol^–1^), air temperature (±0.5°C) and humidity (±5%) (Figure 1B). The CO_2_ control system sensed and assessed the chamber environmental data; these data were later used to regulate the CO_2_ injection process and attaining the desired chamber CO_2_ levels. When the CO_2_ concentration in a chamber exceeded the set concentration, the air was filtered through 1.0 mol mol^–1^ NaOH solution using a pump controlled by a mini-computer. When the humidity of a chamber exceeded the set concentration, the air was filtered through dry calcium carbonate using a separate pump controlled by the mini-computer.

### Plant Sampling

The seedlings were sampled between 10:00 and 11:00 at the 21st day of the experiment. Plant organs were separated into two portions: the first was immersed in liquid-N and then stored at –80°C for later chemical analyses, and the second portion was oven-dried at 105°C for 20 min, and then at 75°C for at least 48 hours. The dried material was used later for different chemical analyses. Fresh leaf area was measured using a leaf area scanning device (Li-3000; Li-Cor Inc., Lincoln, NE, United States).

### Gas-exchange Measurements

After 21 days of growth under different N source and CO_2_ level conditions, we measured gas exchange and chlorophyll fluorescence simultaneously on the first fully developed leaves during the morning (09:00–11:00) using a Li-Cor 6400 infrared gas analyzer (Li-Cor 6400; Li-Cor Inc.). The leaf temperature during measurements was maintained at 25.0 ± 0.5^*o*^C. Leaves were illuminated with a steady red and blue light source at a photosynthetic photon flux density (PPFD) of 1,500 μmol m^–2^ s^–1^ (Yong et al., 2000, 2010). The reference CO_2_ concentrations in the cuvettes matched the treatment CO_2_ concentrations to which samples had been previously subjected (C_*treatment*_), i.e., 280 ± 2.5, 400 ± 2.5 or 550 ± 2.5 μmol mol^–1^. The vapor pressure deficit (Vpdl) was 1.1 ± 0.05 kPa, and the relative humidity was in the range 55–65%. The gas exchange instrument was calibrated each day before the measurements and matched at least twice a day (between the curves). Data were recorded after sample acclimation in the cuvette for at least 15 min.

Two types of curves were plotted: net photosynthesis (*A*_*n*_) *vs*. intercellular CO_2_ concentrations (*C*_*i*_, Supplementary Figure S2), and *A*_*n*_ *vs*. PPFD. Simultaneous measurements of chlorophyll fluorescence and parameters for plotting the A/*C*_*i*_ curves were made on the same leaf using the Li-Cor 6400 infrared gas analyzer. Leaf temperature, PPFD, Vpdl and relative humidity were maintained as indicated above. Prior to measurement, leaves were held in the cuvette at a reference CO_2_ concentration of C_*treatment*_ for at least 10 min. The reference CO_2_ concentration was controlled across a series of C_*treatment*_ values: 200, 150, 100, 50, 400, 600, 800, 1,000, 1,200, and 1,500 μmol mol^–1^. Data were collected after the prevailing CO_2_ had reached a steady state (2–3 min).

### Method for Determining Photosynthetic Parameters of C_3_ and C_4_ Plants

The parameters for the C_3_ wheat plant photosynthesis model were calculated using the equations of Farquhar et al. (1980); Long and Bernacchi (2003), Gao et al. (2018), and Wang et al. (2019).

According to the photosynthesis model that we used for C_4_ maize plants (von Caemmerer and Farquhar, 1999), the rates of phosphoenolpyruvate (PEP) and Rubisco carboxylation (*V*_*p*_ and *V*_*c*_, respectively) are the major determinants of the net CO_2_ assimilation rate. The Rubisco carboxylation rate (*V*_*cmax*_), the maximal rate of PEP carboxylation (*V*_*pmax*_), maximum RuBP-regeneration rate (*J*_*max*_) and CO_2_ concentration in the bundle sheath (*C*_*s*_) for maize were calculated using the following equations. The photosynthetic rate was expressed mathematically as:

(1) $$A=V_{p}-R_{m}-L$$

and

(2) $$A=V_{c}-0.5V_{0}-R_{d}$$

where *R*_*m*_ is the mitochondrial respiration of the mesophyll cells, *L* is the rate of CO_2_ leakage from the bundle sheath into the mesophyll, and *R*_*d*_ is the mitochondrial respiration rate in the light.

(3) $$L=g_{bs}\times \left(C_{s}-C_{m}\right)$$

(4) $$C_{m}=C_{i}-\frac{A}{g_{i}}$$

where *g*_*bs*_ is the bundle sheath conductance for CO_2_, *g*_*i*_ is the mesophyll conductance for CO_2_, *C*_*s*_ is the CO_2_ concentration in the bundle sheath, and *C*_*m*_ is the CO_2_ concentration in the mesophyll cells. *V*_*o*_ is the rate of Rubisco oxygenation:

(5) $$V_{0}=\frac{2\gamma *O}{C_{s}}\times V_{c}$$

where γ^∗^ is one half of the reciprocal of Rubisco specificity (*S*_*c/o*_), and *O* is the oxygen concentration in the bundle sheath cells, which matches the oxygen concentration in the mesophyll cells. By fitting equation (3) to equation (1), and equation (5) to equation (2), we obtained the following expressions:

(6) $$A=V_{p}-R_{m}-g_{bs}\times \left(C_{s}-C_{m}\right)$$

and

(7) $$A=V_{c}\times \left(1-\frac{\gamma *O}{C_{s}}\right)-R_{d}$$

*V*_*p*_ and *V*_*c*_ depend on *V*_*cmax*_, *V*_*pmax*_, *J*_*max*_, the Michaelis constants for O_2_ and CO_2_ (*K*_*o*_, *K*_*c*_ and *K*_*p*_), and the relative specificity of Rubisco (*S*_*c/o*_).

To calculate *V*_*cmax*_ and *V*_*pmax*_, we used the enzyme-limited expressions of *V*_*p*_ and *V*_*c*_:

(8) $$V_{p}=\frac{C_{m}V_{p_{\max}}}{C_{m}+K_{p}}$$

(9) $$V_{c}=\frac{C_{s}V_{c\max}}{C_{s}+K_{p}\left(1+\frac{0}{K_{0}}\right)}$$

By fitting equation (8) to equation (6), and equation (9) to equation (7), we obtained the following expressions:

(10) $$A=\frac{C_{m}V_{p\max}}{C_{m}+K_{p}}-R_{m}-g_{bs}\times \left(C_{s}-C_{m}\right)$$

and

(11) $$A=\frac{C_{s}V_{c\max}}{C_{s}+K_{c}\left(1+\frac{0}{K_{0}}\right)}\times \left(1-\frac{\gamma *O}{C_{s}}\right)-R_{d}$$

In equations (4), (10) and (11), *g*_*i*_, *g*_*bs*_, *R*_*m*_, *K*_*p*_, *K*_*c*_, *K*_*o*_, O, *R*_*d*_ and γ^∗^ were constant parameters at a given temperature [as described by von Caemmerer and Farquhar (1999)], *A* and *C*_*i*_ were measured values, and *C*_*m*_, *C*_*s*_, *V*_*cmax*_ and *V*_*pmax*_ were unknowns. Two pairs of (*A*, *C*_*i*_) (with *C*_*i*_ limited to 40–80 μmol mol^–1^) were then inserted into two sets of equations (5), (8) and (9), following which we obtained six equations and six unknowns (*V*_*cmax*_, *V*_*pmax*_, *C_*s*_1*, *C_*s*_2*, *C_*m*_1* and *C_*m*_2*) using the Matlab software (MathWorks, Natick, MA, United States).

To calculate *J*_*max*_, we used the electron transport limited expressions of *V*_*p*_ and *V*_*c*_:

(12) $$V_{p}=\frac{xJ_{t}}{2}$$

(13) $$V_{c}=\frac{\left(1-x\right)J_{t}}{3\left(1+\frac{7\gamma *O}{3C_{s}}\right)}$$

where *x* is a partitioning factor of electron transport, and *J*_*t*_ is the electron transport rate, given by:

(14) $$J_{t}=\frac{I_{2}+J_{\max}-\sqrt{{\left(I_{2}+J_{\max}\right)}^{2}-4\theta I_{2}J_{\max}}}{2\theta }$$

where *I*_2_ is the total absorbed irradiance, which is a function of the incident irradiance *I*, and *θ* is an empirical curvature factor.

By fitting equation (11) to equation (6), and equation (12) to equation (7), we obtained:

(15) $$A=\frac{xJ_{t}}{2}-R_{m}-g_{bs}\times \left(C_{s}-C_{m}\right)$$

and

(16) $$A=\frac{\left(1-x\right)J_{t}}{3\left(1+\frac{7\gamma^{*}O}{3C_{s}}\right)}-R_{d}$$

*C*_*m*_ was obtained from equation (4). In equations (15) and (16), *x* and γ^∗^ are constants at a given temperature, *A*, *C*_*m*_ and *I* (PPFD) are known values, and *J*_*max*_ and *C*_*s*_ are unknown entities. With two equations and two unknowns, and incorporating the single values of *A* and *I* (PPFD), we obtained the values for *J*_*max*_ and *C*_*s*_.

### Chlorophyll Fluorescence Measurements

Light adapted chlorophyll fluorescence was measured with a Li-Cor 6400 infrared gas analyzer while simultaneously measuring gas exchange, as described above. Steady-state fluorescence (*F*_*s*_) was measured under actinic light. A saturating light pulse (∼8,000 μmol photons m^–2^ s^–1^) was applied for 0.7 s to obtain the maximum fluorescence (*F_*m*_’*). After removing the actinic light and applying 3 s of far-red light, the minimal fluorescence of the light-adapted state (*F_*o*_’*) was obtained. The quantum efficiency of PSII (*Φ_*PSII*_*) and *J*_*t*_ were calculated using equations (17) and (18), respectively, following Genty et al. (1989) and Li et al. (2009):

(17) $$\phi_{PSII}=\frac{F_{m}^{\prime }-F_{s}}{F_{m}^{\prime }}$$

(18) $$J_{t}=\frac{F_{m}^{\prime }-F_{s}}{F_{m}^{\prime }}\times PPFD\times 0.85\times 0.5$$

The central portion of the same leaf (∼70% leaf area) was chosen for measurement of dark-adapted and light-adapted chlorophyll fluorescence parameters using a Fluor imager (CF Imager; Technologia Ltd., Colchester, United Kingdom). The minimum and maximum chlorophyll fluorescence (*F*_*o*_ and *F*_*m*_, respectively) values were determined after full dark adaptation for at least 30 min. *F*_*s*_, *F_*m*_’*, and *F_*o*_’* were obtained as described above. The maximum quantum efficiency of PSII (*F_*v*_/F_*m*_*) was calculated using equation (32) of Genty et al. (1989):

(19) $$F_{v}/F_{m}=\frac{F_{m}-F_{o}}{F_{m}}$$

Photochemical quenching (*qL*) was calculated using equation (20) and non-photochemical quenching (NPQ) was calculated using equation (21), following Kramer et al. (2004).

(20) $$qL=\frac{F_{o}^{\prime }}{F_{s}}\times \frac{F_{m}^{\prime }-F_{s}}{F_{m}^{\prime }-F_{o}^{\prime }}$$

(21) $$NPQ=\frac{F_{m}-F_{m}^{\prime }}{F_{m}^{\prime }}$$

### Calculating Electron Flux to the Photosynthetic Carbon Reduction Cycle [Je(PCR)], and Electron Flux to the Photorespiratory Carbon Oxidation Cycle [Je(PCO)]

The *J*_*t*_ in the photosynthetic carbon reduction and photorespiratory carbon oxidation cycles were expressed as follows (Zhou et al., 2004):

(22) $$Je\left(PCR\right)=4\times \nu_{c}=4\times \frac{A+R_{d}}{1-\frac{\Gamma *}{C_{i}}}$$

(23) $$Je\left(PCO\right)=4\times \nu_{o}$$

### Determination of Free NH_4_^+^ and Soluble Sugar Concentrations

The free NH_4_^+^ in plant tissues was determined according to Balkos et al. (2010) with some modifications. Briefly, plant tissues were desorbed in 10 mM CaSO_4_ for 5 min, and then rinsed with deionized water to remove any extracellular NH_4_^+^. Approximately 0.5 g of fresh material was homogenized with liquid nitrogen; NH_4_^+^ was then extracted in 5 ml of 10 mM formic acid. Supernatants were collected after centrifugation at 10,000 *g* (4°C) for 15 min, transferred to 5-ml polypropylene tubes after filtration through 0.45-μm organic ultra-filtration membranes, and re-centrifuged at 50,000 *g* (4°C) for 10 min. An *O*-phthalaldehyde (OPA) reagent was prepared by combining 200 mM potassium phosphate buffer (equimolar amounts of potassium dihydrogen phosphate and potassium monohydrogen phosphate), 3.75 mM OPA, and 2 mM 2-mercaptoethanol (v/v/v = 1:1:1). Prior to adding 2-mercaptoethanol, the pH was adjusted to 7.0 using 1 M NaOH, and the solution was then filtered through two layers of filter paper. A 10-μl aliquot of tissue extract was mixed with 3 ml of OPA reagent. The color was developed in darkness at 25°C for 30 min before carrying out absorbance measurements at 410 nm using a spectrophotometer (model UV-2401, Shimadzu Corp., Kyoto, Japan).

Soluble sugar concentrations were measured following the method of Wang et al. (2016a). Dry powdered shoot and root samples (0.5 g) were extracted in 80% (v/v) ethanol at 80°C for 30 min. The extracts were later centrifuged at 3000 *g* for 10 min and the supernatants were collected. This extraction procedure was repeated three times to ensure all soluble sugars were extracted. The supernatants were evaporated on china dishes in a hot water bath. Residues were then re-dissolved in 1-3 ml of distilled water and filtered through 0.4-μm film to assay soluble sugars. Concentrations of soluble sugar were measured using the anthrone method. Anthrone sulfuric acid (5 ml) solution (75% v/v) was added to 0.1 ml of supernatant and heated to 90°C for 15 min. Absorbance at 620 nm was measured using a spectrophotometer (model UV-2401, Shimadzu Corp., Kyoto, Japan).

### Statistical Analysis

We found significant effects of N forms (Mix-N, NN, AN) and CO_2_ levels on the measured parameters in wheat and maize using the two-way ANOVA (*P* < 0.05, *n* = 3). Significant pairwise differences between means were identified with Dunnett’s multiple comparisons test (*P* < 0.05). The proportion of variation (%) explainable by each factor was estimated as the total sums of squares. Calculations were performed with SPSS software (SPSS, Inc., Chicago, United States). Graphs were plotted using SigmaPlot 10.0 software (Systat Software, Inc., Chicago, IL, United States).

## Results

### Dry Biomass, Leaf Area and Free NH_4_^+^

**FIGURE 2:**
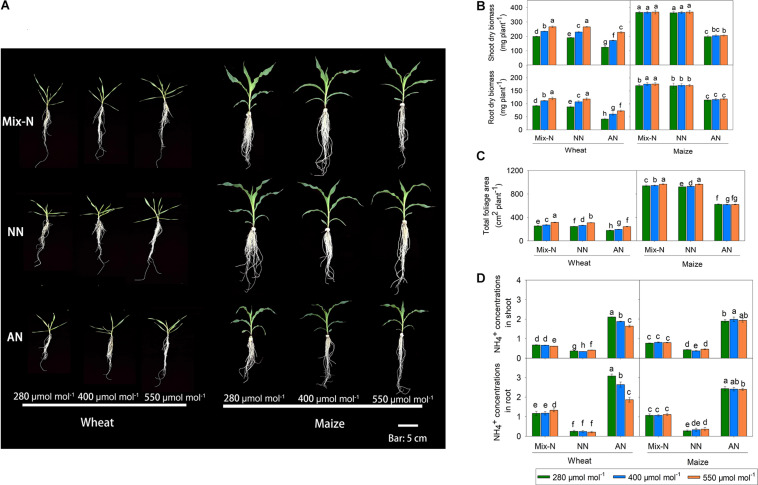
Effects of CO_2_ levels on shoot and root biomass (**A,B**, mg DW plant^–1^), total foliage area (**C**, cm^2^ plant^–1^) and free NH_4_^+^ (**D**, μg g^–1^ FW) of C_3_ wheat and C_4_ maize seedlings after 21 days of treatment with different N-sources. Data are means ± SE (*n* = 3); Lower-case letters indicate a significant difference at *p* < 0.05.

Compared with the Mix-N treatment, AN significantly reduced the shoot and root biomass of both wheat and maize plants (Figure 2). However, with increasing CO_2_ concentration, wheat shoot and root biomass increased significantly, although these biomass parameters did not differ significantly according to CO_2_ levels in maize. Shoot biomass in wheat under AN was reduced by 38%, 27%, and 14% at CO_2_ concentrations of 280, 400 and 550 μmol mol^–1^, respectively (in comparison with the Mix-N treatment). The decreases were larger in maize (46%, 44% and 44% at CO_2_ concentrations of 280, 400, or 550 μmol mol^–1^, respectively) (Figure 2). The AN treatment reduced the total leaf area, where the reduction was again greater in maize than in wheat. With increasing CO_2_ concentrations, the total foliage area of wheat increased significantly, but this was not the case for maize. Free NH_4_^+^ concentrations did not differ significantly in either species between the Mix-N and NN treatments with increasing CO_2_ concentration (Figure 2) whereas, in comparison with the other two treatments, the AN increased free NH_4_^+^ in shoots and roots. The concentration of free NH_4_^+^ decreased significantly with increasing CO_2_ concentration in wheat, but not in maize.

Only the N form had significant effects on maize shoot dry biomass; in wheat, the N form, CO_2_ level, and their interaction significantly affected shoot biomass (Table 1). Changes in CO_2_ levels caused a higher proportion of the variance in wheat shoot dry biomass than did changes in N form. Total leaf area in the two species was significantly affected by both N form and CO_2_ level. N form explained a larger proportion of the variance in total leaf area in both species. Alterations in CO_2_ levels explained a much greater proportion of the variance in total leaf area variation in wheat (38%) than in maize (0.5%). In both species, the quantity of free NH_4_^+^ in shoots and roots differed significantly according to the form of N supplied. Interestingly, the CO_2_ level had significant effects on free NH_4_^+^ in tissues of wheat, but not in maize.

**TABLE 1:** *F-*values in two-way ANOVA analysis of biomass, total foliage area and free NH_4_^+^ in newly expanded leaves of C_3_ wheat and C_4_ maize seedlings after 21 days of treatment with different N-sources.

Species	N-form	CO_2_ level	Shoot dry weight		Root dry weight		Total foliage area		Free NH_4_^+^ in shoot		Free NH_4_^+^ in root	
Species	Source	df	Squares sum (×10^3^)	*F-*value	Squares sum (×10^2^)	*F-*value	Squares sum (×10^3^)	*F-*value	Squares sum	*F-*value (×10^2^)	Squares sum	*F-*value (×10^2^)
Wheat	N	2	38.8(38.1)	2758**	279.8(76.5)	1822**	61(61.2)	1824**	23.09(97.1)	337.6**	47.6(91.0)	115.0**
	CO_2_	2	60.5(59.4)	4302**	82.8(22.6)	539**	38(38.1)	1134**	0.24(1.0)	3.5**	1.2(5.3)	2.9**
	N × CO_2_	4	2.3(2.3)	83**	0.3(0.1)	1 ns	0 (0.0)	1 ns	0.40(1.9)	3.3**	3.4(15.1)	4.1**
	Error	40	0.3(0.3)		3.1(0.8)		1(0.7)		0.01(0.1)		0.1(0.4)	
Maize	N	2	321.2(99.3)	3445.6**	372.4(97.9)	1279**	1258(99.2)	42843**	22.50(99.3)	48.0**	40.0(99.6)	71.9**
	CO_2_	2	0.3(0.08)	2.7 ns	1.6(0.4)	5**	6(0.5)	197**	0.01(0.0)	0.0 ns	0.0(0.0)	0.0
	N × CO_2_	4	0.1(0.03)	0.5 ns	0.6(0.1)	1 ns	4(0.3)	61**	0.04(0.2)	0.1**	0.0(0.2)	0.0
	Error	40	1.9(0.58)		5.8(1.5)		1(0.0)		0.09(0.4)		0.1(0.5)	

N refers to the three nitrogen-forms effect; CO_2_ refers to the three CO_2_ levels effect; N × CO_2_ refers to nitrogen-form × CO_2_ levels interaction effects. Values in brackets following the sum of squares for variables indicate the percentage (%) of given variation to total variation. ** indicate significant at 0.01 level, respectively. df refers to the degree of freedom. ns means no significant difference.

### Photosynthesis and Its Related Parameters

**FIGURE 3:**
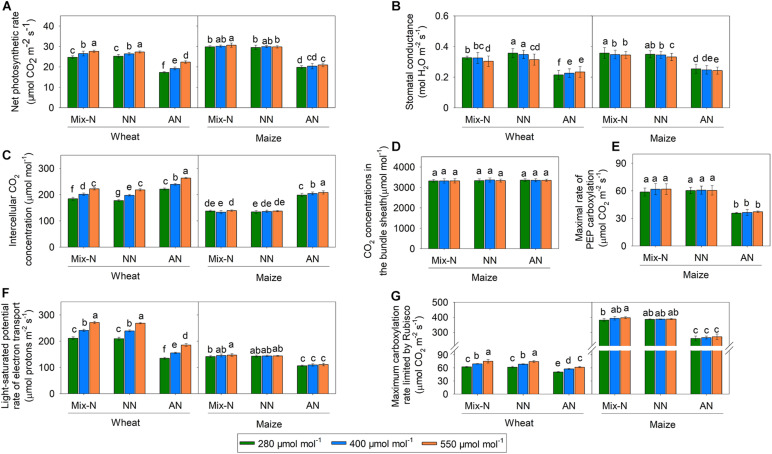
Effects of CO_2_ levels on *P*_*n*_ (**A**, μmol CO_2_ m^–2^ s^–1^), *g*_*s*_ (**B**, mol H_2_O m^–2^ s^–1^), *C*_*i*_ (**C**, μmol mol^–1^), *C*_*s*_ (**D**, μmol mol^–1^), *V*_*cmax*_ (**E**, μmol CO_2_ m^–2^ s^–1^), *V*_*pmax*_ (**F**, μmol CO_2_ m^–2^ s^–1^), and *J*_*max*_ (**G**, μmol protons m^–2^ s^–1^) in newly expanded leaves of C_3_ wheat and C_4_ maize seedlings after 21 days of treatment with different N-sources. Data are means ± SE (*n* = 3); Lower-case letters indicate a significant difference at *p* < 0.05.

Compared with Mix-N and NN, AN treatment reduced the net photosynthetic rate (*P*_*n*_) of both maize and wheat, although the effect was greater in the former species (Figure 3). The *P*_*n*_ of wheat plants growing under 550 μmol CO_2_ mol^–1^ was significantly higher than that of plants grown under 280 and 400 μmol CO_2_ mol^–1^. Conversely, the *P*_*n*_ of maize plants under AN did not differ significantly according to the CO_2_ level. On day 21 of the experiment, the *P*_*n*_ of maize under AN was reduced in comparison with those under the Mix-N treatment, by 34%, 32% and 32% at CO_2_ concentrations of 280, 400, and 550 μmol mol^–1^, respectively. The respective reductions in wheat were 30%, 27 % and 19%.

The *g*_*s*_ values did not differ significantly by CO_2_ concentration, but were significantly reduced by AN treatment compared with the other two treatments, in both species (Figure 3). *C*_*i*_ increased significantly with increasing CO_2_ concentration in wheat, but not in maize. The AN treatment significantly increased *C*_*i*_ in both species in comparison with the other two treatments. On day 21 of the experiment, we found no significant differences in *C*_*s*_ according to either the treatment or CO_2_ concentration. *V*_*pmax*_ did not vary significantly by CO_2_ concentration, but was significantly reduced under AN in comparison with the other two treatments.

The *V*_*cmax*_ and *J*_*max*_ of wheat increased significantly with increasing CO_2_ concentration within the same level of the N form factor, but this was not the case for maize (Figure 3). In comparison with the other two treatments, AN reduced *V*_*cmax*_ and *J*_*max*_ in both species. The reduction in *V*_*cmax*_ associated with AN was larger in maize than in wheat, while the reduction in *J*_*max*_ was smaller in maize than in wheat. On day 21 of the experiment, AN reduced *V*_*cmax*_ in maize in comparison with Mix-N, by 32%, 33% and 32% at CO_2_ concentrations of 280, 400, and 550 μmol mol^–1^, respectively. The respective reductions in wheat were 19%, 17%, and 18%. Moreover, AN reduced the *J*_*max*_ of maize in comparison with Mix-N, by 25%, 25%, and 24% at CO_2_ concentrations of 280, 400, and 550 μmol mol^–1^, respectively. The respective reductions in wheat were 36%, 36%, and 32%.

The *P*_*n*_, *g*_*s*_, *C*_*i*_, *V*_*cmax*_ and *J*_*max*_ varied significantly according to both the N treatment type and the CO_2_ level. The N form accounted for a larger proportion of the variance in these parameters in both species (Table 2). The effect of CO_2_ level was much greater in wheat than in maize. The *V*_*pmax*_ of maize was significantly affected only by the N form. The *C*_*s*_ in maize was not affected by the N form, CO_2_ level, or their interaction, nor by the interaction between pH and the N form.

**TABLE 2:** *F-*values in two-way ANOVA analysis of *P*_*n*_, *g*_*s*_, *C*_*i*_, *C*_*s*_, *V*_*cmax*_, *V*_*pmax*_, and *J*_*max*_ in newly expanded leaves of C_3_ wheat and C_4_ maize seedlings after 21 days of treatment with different N-sources.

Species	N-form	CO_2_ level	*P*_*n*_		*g*_*s*_		*C*_*i*_		*C*_*s*_		*V*_*cmax*_		*V*_*pmax*_		*J*_*max*_	
Species	Source	df	Squares sum (×10^3^)	*F-*value	Squares sum (×10^–3^)	*F-*value	Squares sum (×10^3^)	*F-*value	Squares sum (×10^2^)	*F-*value	Squares sum (×10^3^)	*F-*value	Squares sum (×10^2^)	*F-*value	Squares sum (×10^3^)	*F-*value
Wheat	N	2	532(80.7)	1217**	135(91.2)	732**	205.0(57.7)	2758**			1.75(53.7)	273**			80.1(73.0)	2290**
	CO_2_	2	102(15.4)	232**	3(2.0)	16**	145.3(40.9)	4302**			1.38 (42.1)	214**			28.7(26.1)	820**
	N × CO_2_	4	17(2.5)	18*	6(4.1)	16**	0.4(0.1)	83**			0.01(0.3)	1 ns			0.2(0.2)	3*
	Error	40	9(1.3)		4(2.7)		4.7(1.3)				0.13(3.9)				0.7(0.6)	
Maize	N	2	1108(98.3)	1647**	116(96.7)	1113**	546.3(98.4)	2396**	108(3.4)	0.722ns	184.7(97.0)	826**	70.5(92.1)	253.9**	14.9(94.9)	475**
	CO_2_	2	5(0.4)	7**	2(1.7)	16**	2.4(0.4)	10**	10(0.3)	0.066ns	0.9(0.5)	4*	0.3(0.4)	1.0 ns	0.1(0.9)	4*
	N × CO_2_	4	1(0.1)	1 ns	0 (0.0)	1 ns	2.0(0.4)	4**	28(0.9)	0.094ns	0.3(0.2)	1 ns	0.2(0.2)	0.3 ns	0.0(0.3)	1 ns
	Error	40	13(1.2)		2(1.7)		4.6(0.8)		2987(95.3)		4.5(2.3)		5.5(7.3)		0.6(4.0)	

N refers to the three nitrogen-forms effect; CO_2_ refers to the three CO_2_ levels effect; N × CO_2_ refers to nitrogen-form × CO_2_ levels interaction effects. Values in brackets following the sum of squares for variables indicate the percentage (%) of given variation to total variation. * and ** indicate significant at 0.05 and 0.01 level, respectively. df refers to the degree of freedom. ns means no significant difference.

### Electron Transport Parameters

**FIGURE 4:**
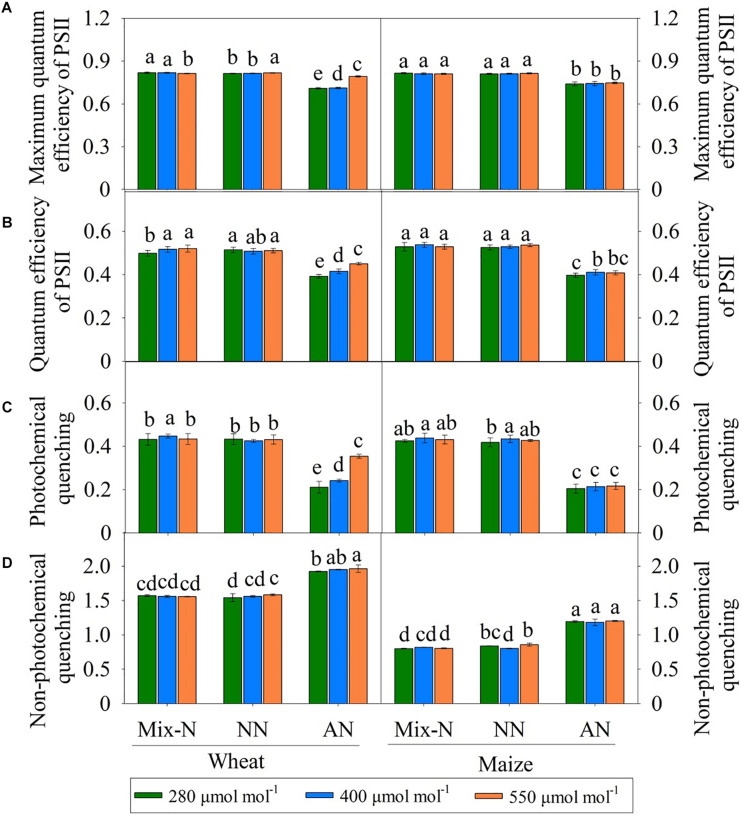
Effects of different CO_2_ levels on *F_*v*_/F_*m*_* **(A)**, *Φ_*PSII*_* **(B)**, NPQ **(C)**, and *qL* **(D)** in newly expanded leaves of C_3_ wheat and C_4_ maize seedlings after 21 days of treatment with different N-sources. Data are means ± SE (*n* = 3); Lower-case letters indicate a significant difference at *p* < 0.05.

Under Mix-N and NN, the F_*v*_/F_*m*_, Φ_*PSII*_ and qL values of the two species did not vary significantly across different CO_2_ concentration (Figures 4A–C). The values of these parameters decreased with increasing CO_2_ concentration in maize plants under AN, but increased in wheat plants as CO_2_ concentrations rose. NPQ increased significantly under AN in comparison with the other treatments, but did not differ significantly by CO_2_ level (Figure 4D). The F_*v*_/F_*m*_, Φ_*PSII*_ and qL values of wheat varied significantly by both N form and CO_2_ concentration, as observed for Φ_*PSII*_ and qL in maize (but not for Fv/Fm). N form explained a larger proportion of the variance in these parameters in both species than CO_2_ concentration (Table 3). CO_2_ level accounted for a larger proportion of the variance in electron transport in wheat (8.9% for F_*v*_/F_*m*_, 5.1% for Φ_*PSII*_ and 5.5% for qL) than in maize (0.0% for F_*v*_/F_*m*_, 0.5% for Φ_*PSII*_ and 0.4% for qL).

**TABLE 3:** *F-*values in two-way ANOVA analysis of *F_*v*_/F_*m*_*, *Φ_*PSII*_*, NPQ and *qL* in newly expanded leaves of C_3_ wheat and C_4_ maize seedlings after 21 days of treatment with different N-sources.

Species	Source	df	*Fv/Fm*		*Φ_*PS*__*II*_*		NPQ		*qL*	
			Squares sum (×10^–3^)	*F-*value	Squares sum (×10^–3^)	*F-*value	Squares sum (×10^–3^)	*F-*value	Squares sum (× 10^–3^)	*F-*value
Wheat	N	2	73(72.3)	11450**	102(86.4)	510**	1768(98.1)	1485**	326(81.7)	1753**
	CO_2_	2	9(8.9)	1369**	6(5.1)	29**	5(0.2)	4*	22(5.5)	117**
	N × CO_2_	4	19(18.8)	1478**	6(5.1)	15**	6(0.5)	3*	47(11.8)	127**
	Error	40	0(0.0)		4(3.4)		24(0.9)		4(1.0)	
Maize	N	2	57(96.6)	478**	189(96.7)	1013**	1671(98.4)	2249**	564(98.8)	2277**
	CO_2_	2	0(0.0)	0 ns	1(0.5)	4*	4(0.2)	5*	2(0.4)	6**
	N × CO_2_	4	0(0.0)	1 ns	1(0.5)	2 ns	8(0.5)	6**	0(0.0)	1 ns
	Error	40	2(3.4)		4(2.1)		15(0.9)		5(0.9)	

*N refers to the three nitrogen-forms effect; CO_2_ refers to the three CO_2_ levels effect; N × CO_2_ refers to nitrogen-form × CO_2_ levels interaction effects. Values in brackets following the sum of squares for variables indicate the percentage (%) of given variation to total variation. ^∗^ and ^∗∗^ indicate significant at 0.05 and 0.01 level, respectively. df refers to the degree of freedom. ns means no significant difference.*

**FIGURE 5:**
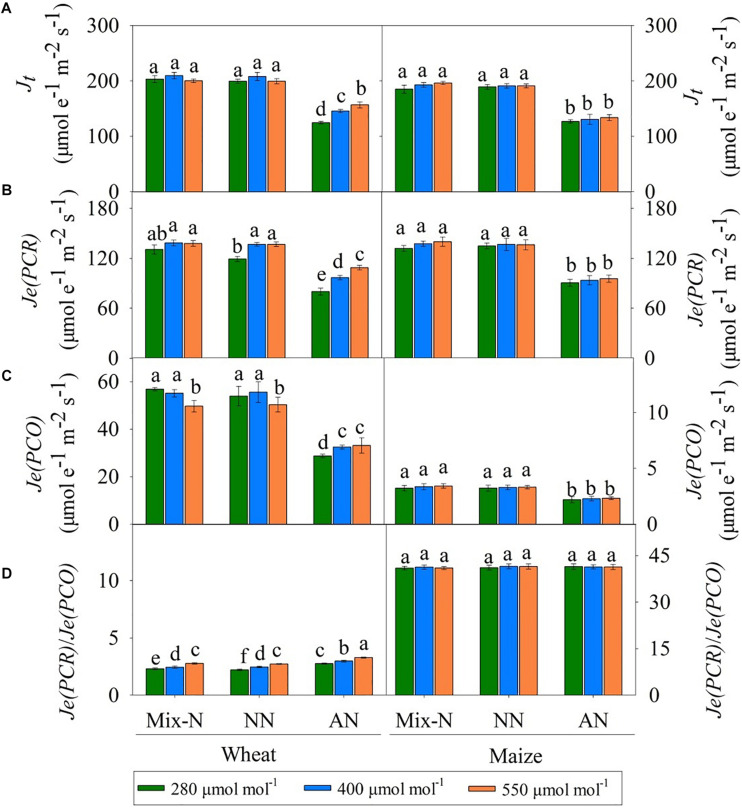
Effects of different CO_2_ levels on *J*_*t*_ **(A)**, *Je(PCR)* **(B)**, *Je(PCO)* **(C)**, and ratio of *Je(PCR)/ Je(PCO)* **(D)** in newly expanded leaves of C_3_ wheat and C_4_ maize seedlings after 21 days of treatment with different N-sources. Data are means ± SE (*n* = 3); Lower-case letters indicate a significant difference at *p* < 0.05.

Under Mix-N and NN, the values of *J*_*t*_ for both species did not differ significantly across different CO_2_ concentration (Figure 5A). However, while the values of *J*_*t*_ for wheat plants under AN increased significantly with increasing CO_2_ concentration, this was not the case for maize. On day 21 of the experiment, AN reduced the *J*_*t*_ values to below those of plants under Mix-N, by 31%, 32% and 32% in maize at CO_2_ concentrations of 280, 400, or 550 μmol mol^–1^, respectively. The respective reductions in wheat were 38%, 30%, and 21%. The *Je(PCR)* values of wheat were higher under CO_2_ concentrations of 400 and 550 μmol mol^–1^ than under a concentration of 280 μmol mol^–1^. CO_2_ level had no significant effect on the *Je(PCR)* values of maize (Figure 5B). In comparison with the other treatments, AN significantly reduced *Je(PCR)* in both species. Under AN, the *Je(PCR)* values of wheat increased significantly with increasing CO_2_ concentration, but this was not the case for maize. When compared to other N form treatments, AN significantly reduced *Je(PCO)* in both species (Figure 5C) on day 21 of the experiment. The *Je(PCR)/Je(PCO)* ratio increased significantly with increasing CO_2_ concentration in wheat. The ratio in maize was unaffected by either CO_2_ level or N form (Figure 5D).

*J*_*t*_, *Je(PCR) Je(PCO)* and the *Je(PCR)/Je(PCO)* ratio were significantly affected by N form and CO_2_ level in both species. N form explained a larger proportion of the variance in these parameters in both species (Table 4). In wheat, 40% of the variance in the *Je(PCR)/Je(PCO)* ratio could be attributed to CO_2_ level, but this factor accounted for only 2.9% of the variance in maize (Table 4).

**TABLE 4:** *F-*values in two-way ANOVA analysis of *J*_*t*_, *Je(PCR)*, *Je(PCO)*, and ratio of *Je(PCR)/ Je(PCO)* in newly expanded leaves of C_3_ wheat and C_4_ maize seedlings after 21 days of treatment with different N-sources.

	Source	df	*J*_*t*_		*Je(PCR)*		*Je(PCO)*		*Je(PCR)/Je(PCO)*	
Species			Squares sum (×10^3^)	*F-*value	Squares sum (×10^3^)	*F-*value	Squares sum	*F-*value	Squares sum	*F-*value
Wheat	N	2	44.5(90.5)	931**	17.6 (79.7)	719**	5885 (90.1)	363.3**	3.29(57.1)	454.2**
	CO_2_	2	1.4(2.9)	30**	3.2 (14.5)	130**	104 (1.6)	6.4**	2.30 (40.0)	318.0**
	N × CO_2_	4	2.3(4.6)	23**	0.8 (3.5)	15**	219 (3.4)	6.7**	0.22 (0.4)	1.5 ns
	Error	40	1.0(1.9)		0.5 (2.2)		323 (5.0)		0.15 (2.5)	
Maize	N	2	43.9 (96.5)	838**	22.2 (95.2)	529**	123 (90.5)	225.9**	0.97(6.1)	1.4 ns
	CO_2_	2	0.4 (0.9)	7**	0.2 (0.9)	5**	0 (1.3)	3.3*	0.46 (2.9)	0.6 ns
	N × CO_2_	4	0.1 (0.3)	1 ns	0.1 (0.3)	1 ns	0 (0.1)	0.1 ns	0.64 (4.1)	0.4 ns
	Error	40	1.0(2.3)		0.8 (3.6)		1 (8.0)		13.64 (86.8)	

N refers to the three nitrogen-forms effect; CO_2_ refers to the three CO_2_ levels effect; N × CO_2_ refers to nitrogen-form × CO_2_ levels interaction effects. Values in brackets following the sum of squares for variables indicate the percentage (%) of given variation to total variation. * and ** indicate significant at 0.05 and 0.01 level, respectively. df refers to the degree of freedom. ns means no significant difference.

### Soluble Sugars

**FIGURE 6:**
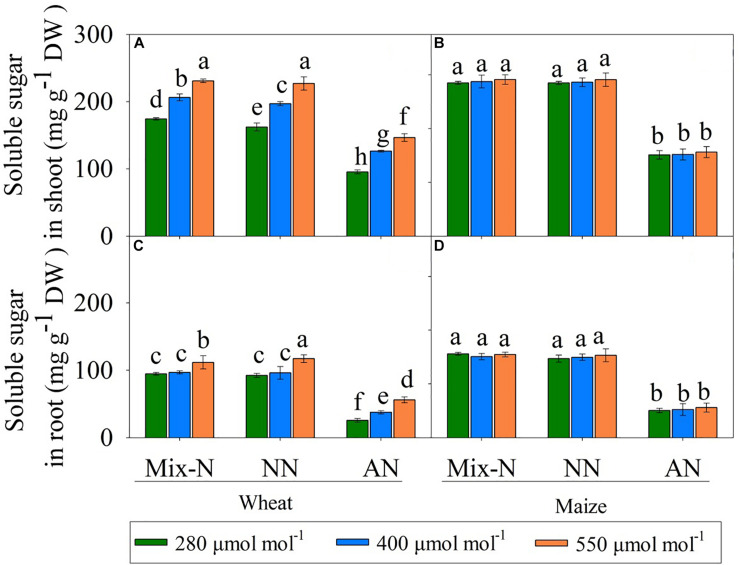
Effects of different CO_2_ levels on soluble sugar concentrations (mg g^–1^ DW) in shoots **(A,B)** and roots **(C,D)** of C_3_ wheat and C_4_ maize seedlings after 21 days of treatment with different N-sources. Data are means ± SE (*n* = 3); Lower-case letters indicate a significant difference at *p* < 0.05.

In comparison with the other N form treatments, AN markedly reduced the soluble sugar concentration in both species. Soluble sugar levels in the shoots and roots of wheat increased with increasing CO_2_ concentration, but this was not the case in maize (Figure 6).

## Discussion

### Increased Atmospheric CO_2_ Concentrations Offset NH_4_^+^-Linked Stress in C_3_ Wheat but Not in C_4_ Maize

Unlike field or pot experiments, hydroponic experiments remove any potential complex interaction between ions and soil particles that might affect nutrient availability, and thus plant growth and development (Conn et al., 2013; Nguyen et al., 2016). We harnessed the hydroponic approach to study the responses of C_3_ wheat and C_4_ maize to different N forms and three levels of CO_2_ concentrations. In previous studies, the most obvious effect of AN was reduced biomass production (Britto and Kronzucker, 2002; Li et al., 2011; Wang et al., 2016b, 2019). In the present study, the Mix-N and NO_3_^–^-fed C_3_ wheat plants produced more dry biomass when the corresponding *C*_*a*_ concentration was increased, but this was not the case for C_4_ maize, corroborating the findings of Leakey et al. (2006). When *C*_*a*_ was low, the leaf-free NH_4_^+^ content was highest in C_4_ maize with NH_4_^+^ as the sole N source (Figure 2); concomitantly under these conditions, we recorded the lowest *P*_*n*_ values for maize (Figure 3).

In C_3_ wheat, AN-induced photosynthetic inhibition was ameliorated by increasing the *C*_*a*_ concentration, but this effect was insignificant in C_4_ maize (Figure 3). Franks et al. (2013) found that change in *C*_*a*_ concentration changes the rates of carboxylation by Rubisco (in C_3_ plants) and PEP carboxylase (in C_4_ plants); each of these enzymes has a crucial limiting step in the photosynthetic pathway. An initial increase in *P*_*n*_ (less pronounced in C_4_ plants) at or above the ambient *C*_*a*_ concentration occurs because of the unique CO_2_-concentrating mechanism associated with C_4_ photosynthesis (Ghannoum et al., 1997; von Caemmerer and Farquhar, 1999). In our study, we further explored (i) the potential limiting factors of *P*_*n*_ when C_3_ and C_4_ plants were grown under condition of NH_4_^+^-N nutrition, and (ii) the way in which changes in *C*_*a*_ concentration affected the potential NH_4_^+^ tolerance of C_3_ and C_4_ plants.

### Impaired Electron Transfer Associated With NH_4_^+^ Inhibited Photosynthesis

Under atmospheric CO_2_ conditions, the carboxylation ability of Rubisco is the key factor limiting C_3_ photosynthesis (Li et al., 2009; Carmo-Silva et al., 2015). *V*_*cmax*_, which represents the apparent Rubisco activity *in vivo* (Long and Bernacchi, 2003), increases with increasing CO_2_ concentration (Jordan and Ogren, 1984). We found that both wheat and maize plants had lower *g*_*s*_ and *V*_*cmax*_ values under AN than under the other two N treatments, but *C*_*i*_ values were elevated under AN (Figure 3). Early CO_2_ enrichment experiments using crops and tree saplings demonstrated that *g*_*s*_ was generally reduced by elevated CO_2_ concentrations; we noted a similar phenomenon in wheat (Figure 3) (Yong et al., 1997; Medlyn et al., 2001). In contrast, the *C*_*i*_ and *C*_*s*_ of maize changed less with increases in *C*_*a*_, regardless of the type of N nutrition. Li et al. (2015) suggested that the increases in *C*_*i*_ may result from decreases in the rate of the photosynthetic dark CO_2_ reduction when *g*_*s*_ is reduced in C_3_ plants.

Under ambient conditions, 44% of the absorbed light at peak PPFD was used for photosynthetic electron transport (25% for CO_2_ fixation, 19% for photorespiration), and the remaining 56% was dissipated by chlorophyll fluorescence and thermal energy generation (Demmig-Adams and Adams, 1992). The balance between photosynthetic electron harvesting and transport within the chloroplasts is important for CO_2_ assimilation based on the Calvin cycle (Demmig-Adams et al., 1989; Fryer et al., 1998; Shikanai, 2011). We found that *Fv/Fm*, *Φ_*PSII*_*, *qL* and *J*_*t*_ were reduced under conditions of NH_4_^+^ nutrition (Figures 4A–C, 5A), indicating that the energy available for CO_2_ assimilation was limited. Similar responses under conditions of NH_4_^+^ nutrition were reported by Johnson et al. (2011), where the photosynthetic electron transport chain was interrupted on the PSII side. The oxygen-evolving complex of PSII may be a direct target of NH_4_^+^, causing a marked decline in photosynthesis (Drath et al., 2008). We found significant reductions in *J*_*max*_, *Φ_*PSII*_* and *J*_*t*_ for both wheat and maize (Figures 3, 4B, 5A); the reductions for maize were especially marked, and led to deficiencies in NADPH and ATP availability for CO_2_ assimilation (Gao et al., 2018). Cousins and Bloom (2003) suggested that NO_3_^–^ assimilation increases linear electron transfer and alleviates the photosynthetic ATP limitation in maize. In NH_4_^+^-fed plants, the inadequate energy supply for CO_2_ carboxylation may be a result of interruptions in the electron transport chain (Wang et al., 2019). With an impaired PSII, plants have a reduced capacity to dissipate excitation energy through *qL*, resulting in a surplus of light energy (Kim and Apel, 2013). We found that NPQ increased in both species (Figure 4D) via a process involving the scavenging of excess light energy through heat dissipation under conditions of NH_4_^+^ nutrition. This finding was consistent with a previous report showing that plants can dissipate excess excitation energy in the form of heat through NPQ when they encountered abiotic stresses (Demmig-Adams and Adams, 1992).

### Higher C_*a*_ Enhanced CO_2_ Assimilation, Which Provided Additional C Skeletons for NH_4_^+^ Assimilation in C_3_ Plants

Under AN, the *J*_*max*_, *Φ_*PSII*_* and *J*_*t*_ values of wheat increased with increasing CO_2_ concentration (Figures 3, 4B, 5A), indicating that the interruption in electron transport can be offset by higher CO_2_ concentration; these parameters did not differ significantly for maize grown under different CO_2_ levels. At low atmospheric CO_2_ levels, Rubisco utilizes both CO_2_ and O_2_ (Edwards et al., 2010). The process of uptaking O_2_ leads to photorespiration, resulting in net losses of ≤40% of photosynthetic carbon under present day CO_2_ levels of 400 μmol mol^–1^ (Andrews and Lorimer, 1978; Sage, 2004; Bloom, 2015). C_4_ photosynthesis suppresses photorespiration by concentrating CO_2_ internally (Andrews and Lorimer, 1978; Ehleringer et al., 1991). Conversely, higher *C*_*a*_ increases the CO_2_ assimilation of C_3_ plants and thereby inhibiting photorespiration; C_4_ plants do not respond in this way (Andrews and Lorimer, 1978). We found that the *C*_*i*_ and *C*_*c*_ values of wheat under AN increased significantly with increased *C*_*a*_, leading to increases in *Je(PCR)*, whereas *Je(PCO)* did not change significantly (Figure 5D). As a result, the *Je(PCR)*/*Je(PCO)* ratio increased with increasing CO_2_ concentration under AN (Figure 5C). These findings indicated that the electron flux to CO_2_ assimilation was increased at higher CO_2_ concentrations, which may compensate for the decrease in electron transport ability seen under AN, thereby sustaining carbon assimilation. In maize, there were no significant differences in *C*_*i*_, *C*_*s*_ or *Je(PCR)/Je(PCO)* by CO_2_ level on day 21 of the experiment (Figures 2, 5D).

Bloom et al. (2010) and Bloom (2015) found that (i) elevated CO_2_ inhibits nitrite (NO_2_^–^) transport into chloroplasts, (ii) the chloroplast stroma compete for reduced ferredoxin (Fdr), and (iii) elevated CO_2_ levels decrease photorespiration, thereby inhibiting shoot NO_3_^–^ assimilation in C_3_ plants under elevated CO_2_ concentrations. In contrast, the first carboxylation reaction in the C_4_ carbon fixation pathway generates ample quantities of malate and NADH in the cytoplasm of mesophyll cells. This explains adequately the CO_2_-independent shoot NO_3_^–^ assimilation in C_4_ plants (Bloom et al., 2010). However, since N assimilation occurs rapidly when NH_4_^+^ is the sole source of N nutrition, an adequate C skeleton supply for NH_4_^+^ assimilation is required to facilitate general physiological homoeostasis under elevated NH_4_^+^ concentrations (Ariz et al., 2013). Therefore, the carbohydrate status of plant tissues has an important role in the transition and adaptation to AN nutrition. A shortage of carbon assimilation for NH_4_^+^-form has been associated with a reduced level of soluble sugars in NH_4_^+^-grown plants (Setien et al., 2013). We found a significant decrease in the soluble sugar concentration in both species, especially in roots, under AN and ambient CO_2_ conditions (Figure 6). With increasing *C*_*a*_, an increase in soluble sugar concentration and a decrease in free NH_4_^+^ concentration occurred in wheat, possibly because of an increase in CO_2_ photosynthetic capacity (Ariz et al., 2010, 2013). Therefore, in wheat, an increased *P*_*n*_ under AN, which was driven by elevated *C*_*a*_ levels, increased the supply of carbon skeleton for NH_4_^+^ assimilation, which in turn reduced the NH_4_^+^ concentrations and thereby ameliorating the NH_4_^+^ stress.

## Conclusion

In conclusion, under ambient CO_2_ conditions and AN nutrition, electron transport was reduced in both the C_3_ wheat and C_4_ maize plants, leading to a suppression of photosynthetic carbon assimilation. In wheat growing under elevated atmospheric CO_2_ concentrations (*C*_*a*_), increased *C*_*i*_ and *C*_*c*_ values improved electron flux to CO_2_ assimilation rather than to photorespiration, thus sustaining photosynthesis and alleviating NH_4_^+^-induced stress. In contrast, elevated *C*_*a*_ had a negligible effect on *C*_*i*_ and *C*_*s*_ in maize and, consequently, minor effects on photosynthesis. Therefore, future increases in atmospheric *C*_*a*_ should provide C_3_ plants with more opportunities to use NH_4_^+^ rather than relying on NO_3_^–^ as a source of N fertilizers for crop production. Analyses using molecular biology and mutants to explain the possible physiological mechanisms in NH_4_^+^ tolerance of crop cultivars.

## Data Availability Statement

All datasets generated for this study are included in the manuscript/Supplementary Material.

## Author Contributions

FW and XH conceived the original screening and research plans. FW, JG, and XH supervised the experiments. FW performed most of the experiments, conceived the project, and wrote the article with salient contributions from all the authors in specific areas. FW, JG, JY, QW, JM, and XH supervised and completed the writing. All authors contributed to the article and approved the submitted version.

## Conflict of Interest

The authors declare that the research was conducted in the absence of any commercial or financial relationships that could be construed as a potential conflict of interest.

## Abbreviations

Γ^∗^: the CO2 compensation point related to Ci
φ: apparent quantum yield
Φ PSII: quantum efficiency of PSII
Ca: atmospheric CO2 concentration
Cc: chloroplastic CO2 concentration
CE: carboxylation efficiency
Ci: intercellular CO2 concentration
Cm: CO2 concentrations in the mesophyll cells
Cs: CO2 concentrations in the bundle sheath
C treatment: the CO2 concentration treatment of each sample
FACE: free-air CO2 enrichment
Fm: the maximum chlorophyll fluorescence with dark-adaptation
Fm’: the maximum fluorescence with light-adaptation
Fo: the minimum chlorophyll fluorescence with dark-adaptation
Fo’: the minimum chlorophyll fluorescence with light-adaptation
Fs: steady state fluorescence with light-adaptation
Fv/Fm: maximum quantum efficiency of PSII
gm: mesophyll conductance
gs: stomatal conductance
Ja: alternative electron flux
Jcmax: maximum carboxylation rates limited by RuBP regeneration
Je(PCO): electron fluxes to photosynthetic carbon oxidation
Je(PCR): electron fluxes to photorespiratory carbon reduction
Jmax: maximum electron transport rates
LHC: light-harvesting complex
Mix-N: a mixture of both NO3– and NH4+ source
NPQ: non-photochemical quenching
NUE: nitrogen use efficiency
OTC: open-top chambers
Pn: net photosynthetic rate
PPFD: photon flux intensity
PSII: photosystem II
PVC: polyvinyl chloride
qL: photochemical quenching
Rd: mitochondrial respiration rate in the light
ROS: reactive oxygen species
Rubisco: ribulose-1,5-bisphosphate carboxylase/oxygenase
vc: the carboxylation rate
Vcmax: maximum carboxylation rate limited by Rubisco
vo: the oxygenation rate
Vpdl: the vapor pressure deficit
Vpmax: the maximal rate of PEP carboxylation
wc: the potential Rubisco carboxylation rate
wj: the potential RuBP regeneration rate
wp: the potential triose-phosphate utilization rate.
