# Supplementary material for: Higher Atmospheric CO2 Levels Favor C3 Plants Over C4 Plants in Utilizing Ammonium as a Nitrogen Source
Source: Front Plant Sci. 2020 Dec 2;11:537443. doi: 10.3389/fpls.2020.537443 (PMC7738331; doi:10.3389/fpls.2020.537443)
Supplement: Supplementary file 1 [file Data_Sheet_1.docx]

Supplementary Material


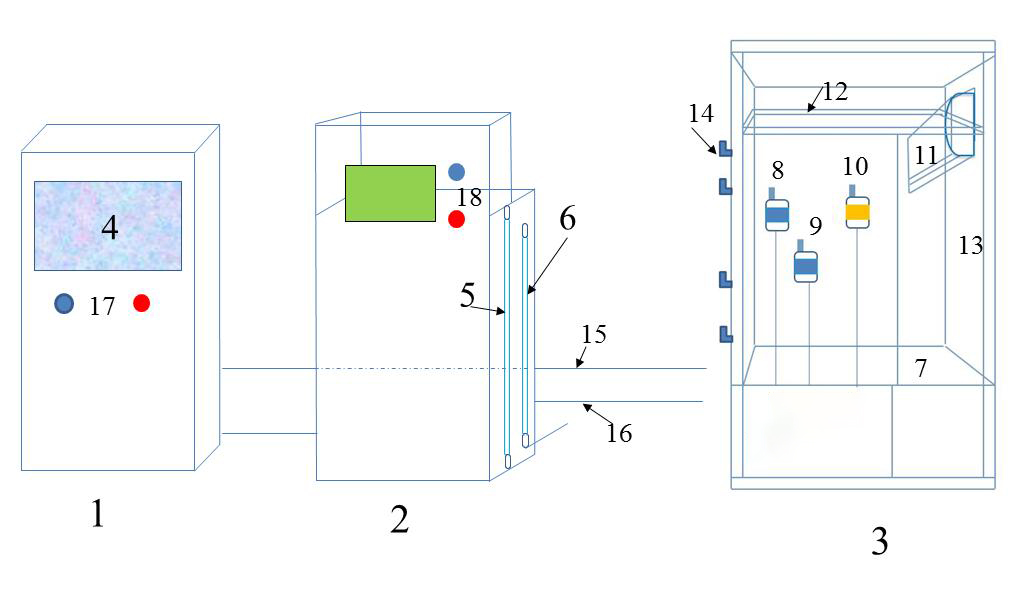


**Fig. S1 |** The automatically controlled-environment facility consists of the CO_2_ control system equipment (1), the CO_2_ generator (2) and nine chambers (3). 4, the liquid crystal display (LCD) screen; 5, Na_2_CO_3_ feeding inlet; 6, H_2_SO_4_ feeding inlet; 7, polyvinyl chloride (PVC) for the floor; 8, the outside temperatures and humidity sensors; 9, the inside temperatures and humidity sensors; 10, the inside CO_2_, sensors; 11, the temperature control system; 12, CO_2_ pipes; 13, toughened glass (10mm, Transmittance: 99%); 14, doorknob of the chamber; 15, signal lines connected the sensors to the control system equipment; 16, CO_2_ pipes connected the chambers to the CO_2_ generator. 17, power switch for the CO_2_ control system equipment; 18, power switch for the CO_2_ generator.


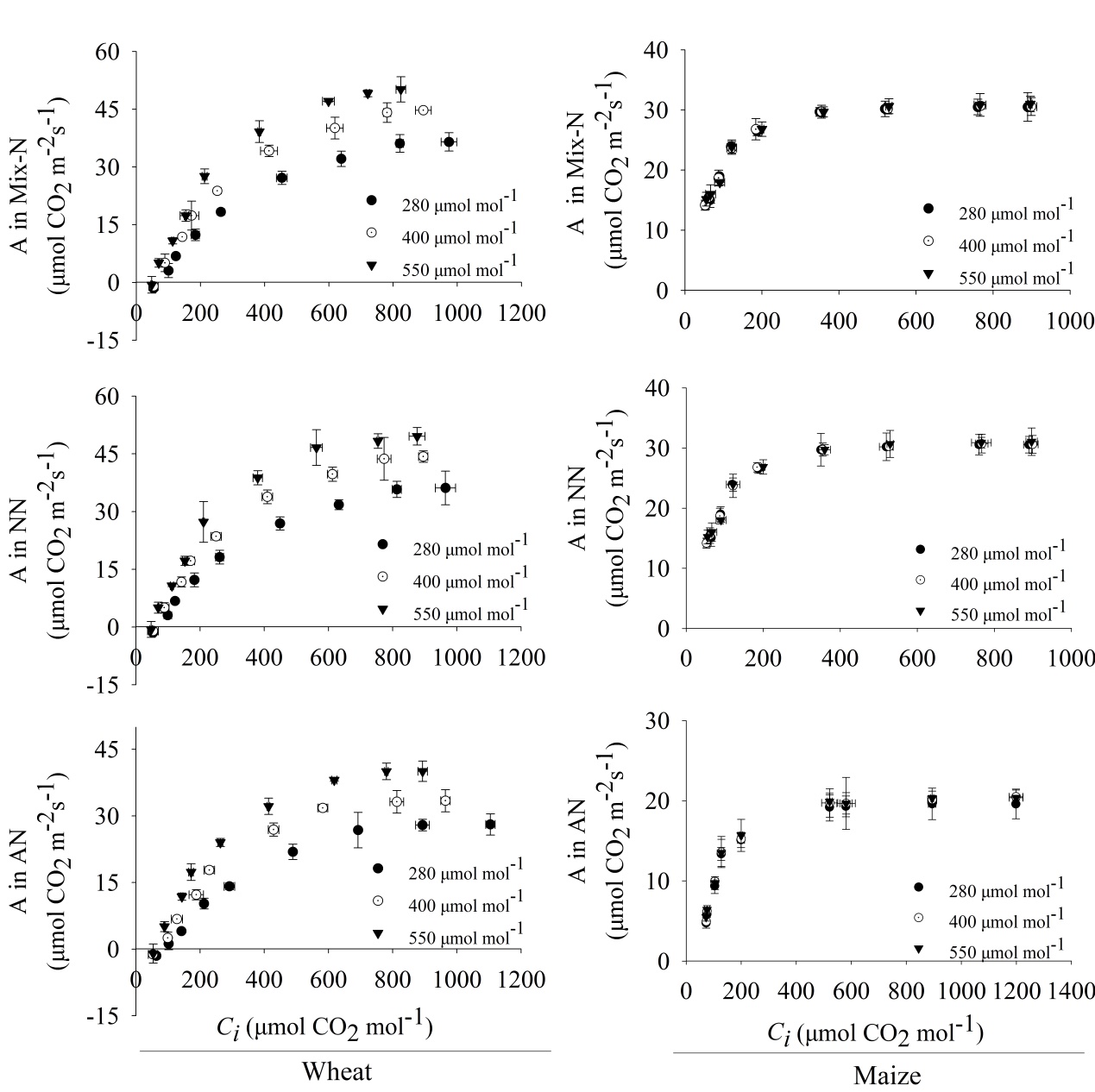


**Fig. S2 |** Effects of different CO_2_ levels on *A/Ci* curves in newly expanded leaves of C_3_ wheat and C_4_ maize seedlings after 21 days of treatment with different N-sources. Data are means ± SE (*n* = 3). NN refers to NO_3_^–^ as a N source; AN refers to NH_4_^+^ as a N source; Mix-N refers a mixture of NO_3_^–^ and NH_4_^+^.
